# Comparison of the Toxicological Effects of Pesticides in Non-Tumorigenic MCF-12A and Tumorigenic MCF-7 Human Breast Cells

**DOI:** 10.3390/ijerph19084453

**Published:** 2022-04-07

**Authors:** Lucia Coppola, Sabrina Tait, Enrica Fabbrizi, Monia Perugini, Cinzia La Rocca

**Affiliations:** 1 Center for Gender-Specific Medicine, Italian National Institute of Health, 00161 Rome, Italy; lucia.coppola@iss.it (L.C.); sabrina.tait@iss.it (S.T.); 2 Department of Physiology and Pharmacology V. Erspamer, Sapienza University of Rome, 00185 Rome, Italy; 3 Pediatric Departmental Simple Operative Unit, Civitanova Marche Hospital, ASUR Marche Area Vasta n. 3, 62100 Macerata, Italy; enrica.fabbrizi@libero.it; 4 Faculty of Bioscience and Technology for Food, Agriculture and Environment, University of Teramo, 64100 Teramo, Italy; mperugini@unite.it

**Keywords:** pesticides, herbicides, endocrine disruptors, nuclear receptors, in vitro model, mammary gland development, idiopathic premature thelarche, MCF-7, MCF-12A

## Abstract

Humans are exposed to residues of organophosphate and neonicotinoid pesticides, commonly used in agriculture. Children are particularly vulnerable and, among possible adverse outcomes, the increased incidence of premature mammary gland development (thelarche) has raised concern. We evaluated the toxicological effects of chlorpyrifos (CPF), imidacloprid (IMI) and glyphosate (GLY) at exposure concentrations occurring in children on the tumorigenic MCF-7 and non-tumorigenic MCF-12A breast cell lines, as representative of the target organ model, assessing cytotoxicity, apoptosis, necrosis, intracellular reactive oxygen species (ROS) and ATP levels, 17β-estradiol secretion and gene expression of nuclear receptors involved in mammary gland development. The pesticides decreased cell vitality in MCF-7 and cell proliferation in MCF-12A cells. ATP levels were decreased in MCF-7 cells by pesticides and apoptosis was increased in MCF-12A cells only by GLY (2.3 nM). ROS production was decreased by pesticides in both cell lines, except IMI (1.6 nM) in MCF-7 cells. Endocrine disrupting activity was highlighted by induction of 17β-estradiol secretion and modulation of the gene expression of estrogen alpha and beta, progesterone, androgen, and aryl hydrocarbon receptors in both cell lines. The use of MCF-7 and MCF-12A cells highlighted dissimilar modes of action of each pesticide at low human relevant concentrations.

## 1. Introduction

Several pesticides are commonly used in agriculture to protect crops [1]. Human exposure to pesticide residues and other environmental pollutants is of concern for human health, since they can affect reproductive, neurological, cardiovascular and endocrine systems. Increasing evidence reported possible associations with male and female infertility, neurological and metabolic diseases and cancer [2,3]. To reduce the impact of pesticides exposure, the European Commission and regulatory agencies have promoted and implemented measures for a sustainable use of pesticides since 2009 [4]. Moreover, a guidance on the criteria to be considered in the assessment of endocrine disrupting properties of pesticides has been recently developed by the European Food Safety Authority (EFSA) and the European Chemicals Agency (ECHA) [5].

As endocrine disrupting chemicals (EDCs), pesticides can derange the hypothalamic–pituitary–gonadal axis by perturbing hormone secretion, which may affect the reproductive female system with adverse effects on ovarian and uterine function, menstrual cycle and timing of puberty [6,7,8,9]. Indeed, children exposure to pesticides has been associated with premature development of the reproductive system, including premature thelarche, the early breast development before the age of 8 years, whose incidence has increased worldwide in the last decades [10,11]. The pubertal breast development is mainly controlled by ovarian estrogen and progesterone hormones, regulating the ductal morphogenesis and the epithelial growth of the mammary gland through their respective estrogen receptors alpha and beta (ERα, ERβ), and the progesterone receptor (PgR) [12]. However, other nuclear receptors (NRs) are also involved, such as the androgen receptor (AR), which has a compensatory role in mammary gland development during puberty, counteracting the extent of cell proliferation, ductal extension and buds morphology [13] and the aryl hydrocarbon receptor (AhR), which is not strictly required for breast development [14], but due to its cross-talk with ERs, is relevant as a regulator of cell proliferation [15].

Among the most widely used pesticides, several pieces of evidence support the organophosphates chlorpyrifos (CPF) and glyphosate (GLY), as well as the neonicotinoid imidacloprid (IMI), as risk factors able to alter mammary gland development.

CPF may act as a weak estrogenic compound affecting ERs expression [16,17], also displaying anti-androgenic, thyroid and AhR agonistic activities [18,19,20]. In in vitro studies, CPF induced cell proliferation in MCF-7 and MDA-MB-231 human breast cell lines by a mechanism involving ERα activation, at least in ER-responsive cells [21,22]. Administration of CPF to adult female rats at 0.01 mg/kg body weight (bw)/day increased the number of mammary gland ducts, and increased cell proliferation as well as PgR expression, but decreased serum estradiol and progesterone levels at a higher concentration (1 mg/kg bw/day) [23]. Furthermore, CPF exposure of adult female rats to 0.1 and 2.5 mg/kg bw/day altered mammary gland morphology, increasing ductal thickness and branching as well as bud diameter and number [24]. Promotion of breast tubulogenesis through the activation of the AhR pathway was also observed in MCF-7 treated with CPF [25].

Recently, IMI was also demonstrated to trigger ER-mediated estrogenic activity, as shown in the MCF-7 transfected MELN cells [26]. Administration of 20 mg/kg bw/day IMI to adult female rats decreased progesterone serum levels, probably due to an alteration in GnRH release [27]. So far, no in vivo studies were performed to investigate IMI adverse effects on mammary gland development.

GLY displayed a weak ER agonism in a transfected epithelial mammary gland cell line (T47D-KBluc cells) [28] and promoted proliferation in MCF-7 cells increasing ERα gene expression via a ligand-independent mechanism, but only at high concentrations [6]. Furthermore, GLY may also act as an inhibitor of aromatase [26]. At the mammary gland level, the early postnatal administration of a GLY-based herbicide formulation (GLY at 2 mg/kg bw) increased the hyperplasia of ducts and altered the stroma morphology; furthermore, it induced ERα and PgR gene expression [29].

Overall, most of the studies did not investigate adverse effects at environmentally relevant concentrations. Furthermore, limited evidence is available from human studies. In this context, the project “Integrated approach to evaluate children agricultural pesticide exposure and health outcome” (PEACH Project) aims to investigate the possible association between pesticide exposure and idiopathic premature thelarche (IPT) in girls by a case-control study and an in vitro study assessing potential toxicological adverse effects of mainly used pesticides [30]. The present study, within the PEACH Project, compares the toxicological effects of CPF, IMI and GLY at real exposure concentrations occurring in children, as detected in urine samples in biomonitoring studies [31,32,33,34,35], in two human mammary gland cell lines: MCF-7, a commonly used tumorigenic cell line, and MCF-12A, a non-tumorigenic cell line more realistically reflecting normal physiological conditions. For this purpose, a battery of tests was performed, including assessment of cell vitality and proliferation, apoptosis and necrosis, reactive oxygen species (ROS) and ATP intracellular amount, 17β-estradiol (E2) secretion and gene expression of NRs involved in mammary gland development (i.e., ERα, ERβ, AR, AhR and PgR).

## 2. Materials and Methods

### 2.1. Chemicals

All reagents of analytical grade were purchased from Sigma-Aldrich (Milan, Italy). Chlorpyrifos (CPF; CAS no. 2921-88-2, purity ≥ 98.0%), imidacloprid (IMI; CAS no. 138261-41-3, purity ≥ 98.0%) and glyphosate (GLY; CAS no. 1071-83-6, purity ≥ 98.0%) stock solutions were prepared in dimethyl sulfoxide (DMSO) for CPF (12 mM) and IMI (16 mM), whereas GLY was dissolved in water (23 mM). Such concentrations were chosen to be conveniently ten-fold diluted in cell culture medium to obtain selected treatment concentrations, as described below.

### 2.2. Cell Culture

A MCF-7 (HTB-22) cell line was purchased from ECACC (Salisbury, UK) and grown in phenol red free-DMEM medium (Gibco, Grand Island, NY, USA), supplemented with 10% fetal bovine serum (Gibco), 100 U/mL penicillin/streptomycin and 2 mM L-glutamine (Gibco).

MCF-12A (CRL-10782) cells were purchased from ATCC (Manassas, VA, USA) and grown in phenol red free-DMEM/F12 medium containing 5% equine serum (ES), 20 ng/mL epidermal growth factor, 0.5 μg/mL hydrocortisone, 0.1 μg/ mL cholera toxin, and 10 μg/mL insulin.

Both cell lines were maintained in a humidified Steri-Cult 200 Incubator (Forma Scientific, Marietta, OH, USA) at 37 °C and 5% CO_2_.

### 2.3. Cell Viability and Proliferation

The colorimetric MTS assay (Cell Titer 96 Aqueous One Solution reagent; Promega, Madison, WI, USA) and the fluorimetric CyQuant^®^ assay (CyQuant^®^ Direct Cell proliferation Assay; Life Technologies, Paisley, UK) were performed in MCF-7 and MCF-12A cells to determine, respectively, cell vitality and proliferation. According to manufacturers’ protocols, for each assay, 10,000 cells/well were plated in 96 flat-bottomed multiwells and incubated overnight in a humidified incubator at 37 °C to permit cell adhesion. Medium was then replaced with fresh medium with five added ten-fold serial dilutions of CPF (120 pM–1.2 µM), IMI (160 pM–1.6 µM) and GLY (230 pM–2.3 µM) or with medium alone. Cells treated with medium alone were considered as control cells. Experiments were performed in triplicated wells, incubating cells for 72 h at 37 °C. Pesticide stock solutions were diluted with culture medium immediately before use; since the DMSO final concentration did not exceed 0.01% (*v*/*v*) being not toxic [36], we did not include solvent control cells in the design. For each pesticide, the range of concentrations used was established starting from the mean exposure levels detected in urine samples of children and reported in biomonitoring studies, i.e., CPF 12 nM, IMI 16 nM and GLY 23 nM [31,32,33,34,35]. After incubation, 20 μL of MTS reagent or 100 μL (equal volume as culture media) of 2X CyQuant Detection Reagent were added to each well, incubating for 60 min at 37 °C. The Victor 3 Multilabel Reader (PerkinElmer, MA, USA) was used to read absorbance at 490 nm (MTS assay) or fluorescence from the bottom with a green filter (CyQuant assay), setting control cells as 100% viable. Three independent experiments were performed for each assay and the GraphPad Prism v 5.01 software (GraphPad Software Inc., La Jolla, CA, USA) was used to derive and visualize the best curve fit for each assay.

### 2.4. Apoptosis-Necrosis Assay

The RealTime-Glo™ Annexin V Apoptosis and Necrosis Assay kit (Promega, Madison, WI, USA) was used following the manufacturer’s protocol. About 10,000 MCF-7 and MCF-12A cells/well were seeded on 96-well solid white bottom microplates. The next day, medium was removed and cells were treated with five ten-fold serial dilutions of CPF (120 pM–1.2 µM), IMI (160 pM–1.6 µM), GLY (230 pM–2.3 µM) or with medium alone as a control, also adding 100 μL of kit’s reagents mix solution. Plates were read for both luminescence and fluorescence (485–535 nm) signals by a Victor 3 Multilabel Reader each hour for the first 8 h and then at 24 h of exposure, normalizing values to control cells’ readings at each incubation time. The assay was performed in three independent experiments; GraphPad Prism v 5.01 (GraphPad Software Inc., San Diego, CA, USA) was used to visualize the results.

### 2.5. ATP Levels Assessment

The Mitochondrial ToxGlo™ Assay (Promega, Madison, WI, USA) was performed to assess intracellular ATP levels. According to the manufacturer’s protocol, about 10,000 MCF-7 or MCF-12A cells/well were plated in 96 flat-bottomed multiwells and incubated overnight at 37 °C to permit cell adhesion. The experimental design (i.e., cell treatment and pesticide concentrations) was the same as for the cell viability/proliferation assessment (see Section 2.3). After 72 h incubation, 100 μL of ATP Detection Reagent was added to each well stirring at 500 rpm for 5 min; luminescence was then read on a Victor 3 Multilabel Reader (PerkinElmer, Waltham, MA, USA), normalizing readings with respect to control cells set at 100%. Three independent experiments were performed and GraphPad Prism v.5.01 (GraphPad Software Inc., San Diego, CA, USA) was used to visualize the results.

### 2.6. Reactive Oxygen Species Assay

The ROS Detection Assay Kit (BioVision, Milpitas, CA, USA) was used to assess intracellular ROS levels following the manufacturer’s protocol. Briefly, about 10,000 MCF-7 or MCF-12A cells/well were plated in 96 flat-bottom microplates and incubated overnight allowing adhesion.

The next day, medium was removed and 100 μL/well of ROS Assay Buffer was added to wash the cells; after buffer removal, 100 μL/well of ROS Assay Label 1X was added, incubating for 45 min at 37 °C. The ROS label solution was then removed and cells were treated for 24 h with CPF (at 1.2–12–120 nM), IMI (at 1.6–16–160 nM), GLY (at 2.3–23–230 nM) or with medium alone as a control. In the range of concentrations tested, the intermediate ones correspond to mean exposure levels detected in urine samples of children [31,32,33,34,35]. H_2_O_2_ 100 μM was used as a positive control. At the end of treatment, plates were read by the Victor 3 Multilabel Reader (PerkinElmer, Waltham, MA, USA) measuring fluorescence from the bottom (485 nm ex–535 nm em), setting control cells as 100%. The assay was repeated in three independent experiments.

### 2.7. Cell Treatment

MCF-7 and MCF-12A cells were plated in culture flasks and incubated overnight at 37 °C, 5% CO_2_ and 90% humidity to permit cell adhesion. Then, cells were treated with CPF (1.2, 12 or 120 nM), IMI (1.6, 16 or 160 nM), GLY (2.3, 23 or 230 nM) or with medium alone as a control for 72 h at 37 °C. Three independent experiments were performed for each pesticide, at different replication passages from thawing. At the end of incubation, supernatants and cell monolayers were collected and stored at −80 °C for ELISA and Real-time PCR analyses, respectively.

### 2.8. Estradiol ELISA Assay

MCF-7 and MCF-12A culture supernatants were assessed for 17β-estradiol (E2) secretion by the Estradiol parameter assay kit (R&D Systems, Minneapolis, MN, USA) according to the manufacturer’s protocol. By the provided E2 standard solution, six three-fold serial dilutions (12.3 pg/mL–3000 pg/mL) were prepared and assessed along with the samples in duplicated wells. Absorbance was read at 450 nm by the Victor 3 Multilabel Reader (PerkinElmer, Waltham, MA, USA); unknown E2 concentrations in samples were derived from the E2 standard curve by the GraphPad Prism v.5.01 software (GraphPad Software Inc., San Diego, CA, USA).

### 2.9. Real-Time PCR

Total RNA content was extracted from MCF-7 and MCF-12A cell pellets using the Norgen RNA kit (Norgen, Thorold, ON, Canada) according to the manufacturer’s protocol. A Nabi Nano Spectrophotometer (MicroDigital Co., Ltd., Seongnam, Korea) was used to determine RNA quantity.

An aliquot of total RNA (1 μg) from each sample was reverse transcribed to cDNA using the SensiFAST cDNA Synthesis Kit (Bioline Reagents Ltd., London, UK). Specific primers for the selected panel of genes (ERα, ERβ, AR, AhR, and PgR), as well as for GAPDH and ACTB as reference genes, were designed through the web application Primer BLAST (www.ncbi.nlm.nih.gov/tools/primer-blast (accessed on 16 March 2022)) and purchased from Invitrogen (Thermo Fisher Scientific, Waltham, MA, USA). Primer sequences are listed in Table 1. Reactions were run in duplicate on 96-well PCR plates using the SensiMix SYBR kit (Bioline). The thermal program was as follows: 1 cycle at 95 °C for 10 min; 40 cycles at 95 °C for 15 s, 58 °C for 30 s and 72 °C for 1 min; 1 dissociation cycle from 55 to 95 °C (30 s/°C) to verify amplification products. Threshold cycles (Ct) were obtained by the LineGene 9600 PCR V.1.0 software (Bioer) and ΔΔCt values were calculated using control cells as calibrators and the geometric mean of housekeeping genes’ Ct values as a normalizer.

### 2.10. Statistical Analysis

The statistical analysis was performed with the JMP 10 Software (SAS Institute Inc., Cary, NC, USA). Statistical differences among treated and control cells were evaluated by the analysis of the variance (ANOVA) followed by the post-hoc Dunnett’s test where appropriate. Results with *p* < 0.05 were considered significant. Data are presented as mean ± standard error mean (SEM) values.

## 3. Results

### 3.1. Cell Viability and Cell Proliferation

**Figure 1:**
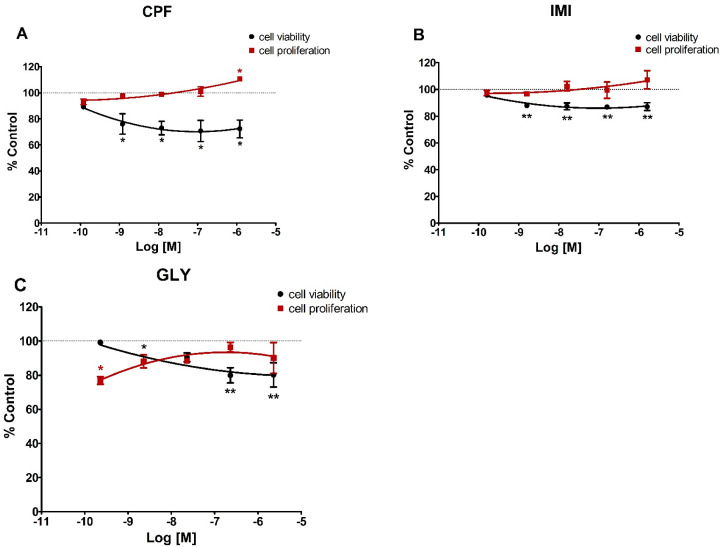
Dose-response curves for cell viability (black) and cell proliferation (red) assays in MCF-7 cells treated for 72 h with CPF (**A**), IMI (**B**) or GLY (**C**). Values are means ± SEM of three independent experiments with control cells set at 100%. Asterisks indicate statistically significant differences with respect to control cells: * *p* < 0.05; ** *p* < 0.01.

The three pesticides under study determined different cytotoxic outcomes, mostly decreasing cell vitality in MCF-7 and cell proliferation in MCF-12A cells. In particular, in MCF-7 cells (Figure 1), CPF and IMI significantly decreased cell viability at all concentrations tested except the lowest (120 and 160 pM, respectively), whereas GLY induced a significant decrease in cell viability at 2.3 nM, 230 nM and 2.3 µM concentrations. The pesticides did not significantly affect cell proliferation in this cell line except for CPF at the highest concentration (1.2 µM) and GLY at the lowest (230 pM).

**Figure 2:**
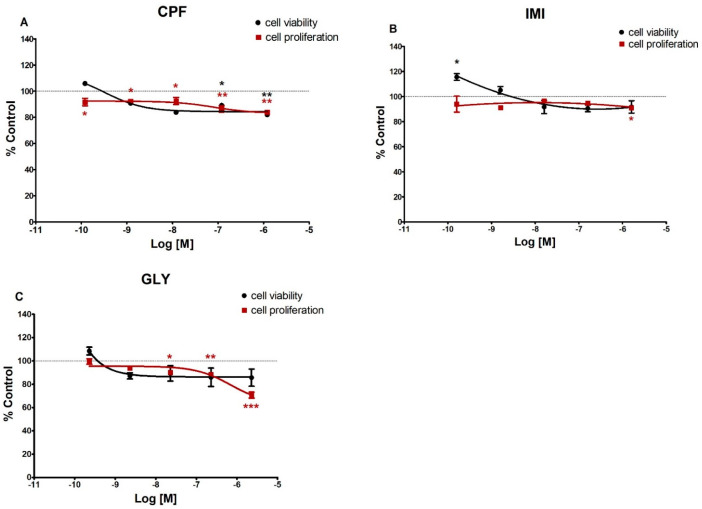
Dose-response curves for cell viability (black) and cell proliferation (red) assays in MCF-12A cells treated for 72 h with CPF (**A**), IMI (**B**) or GLY (**C**). Values are means ± SEM of three independent experiments with control cells set at 100%. Asterisks indicate statistically significant differences with respect to control cells: * *p* < 0.05; ** *p* < 0.01; *** *p* < 0.001.

In MCF-12A (Figure 2), cell viability was decreased only by CPF at the two highest doses of (120 nM and 1.2 µM), whereas IMI at 160 pM induced a significant increase of cell vitality. Conversely, cell proliferation was significantly reduced by CPF at all tested concentrations, by IMI at the highest dose (1.6 µM) and by GLY at the higher doses (23 nM, 230 nM and 2.3 µM).

### 3.2. Apoptosis and Necrosis Time Course

CPF, IMI and GLY did not induce statistically significant differences in apoptotic and necrotic signals in MCF-7 cells, at all tested concentrations (Figure S1).

**Figure 3:**
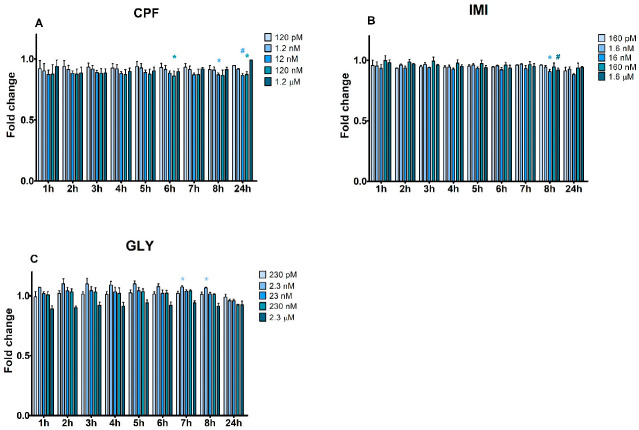
Apoptotic signals evaluated by Annexin V assay in MCF-12A cells treated with CPF (**A**), IMI (**B**) and GLY (**C**) or medium alone as a control for 24 h. Data are mean fold change values of three independent experiments. Asterisks and hashtags indicate statistically significant differences with respect to control cells: * *p* < 0.05; # *p* < 0.01.

In MCF-12A (Figure 3), GLY was the only pesticide increasing apoptosis at 7 and 8 h at 2.3 nM. Otherwise, CPF and IMI significantly decreased the apoptotic signal: CPF at 6 h (120 nM), 8 h (12 nM) and 24 h (12 nM and 120 nM) and IMI at 8 h (16 nM and 1.6 µM). No significant necrotic effect was observed (Figure S2).

### 3.3. ATP Levels

**Figure 4:**
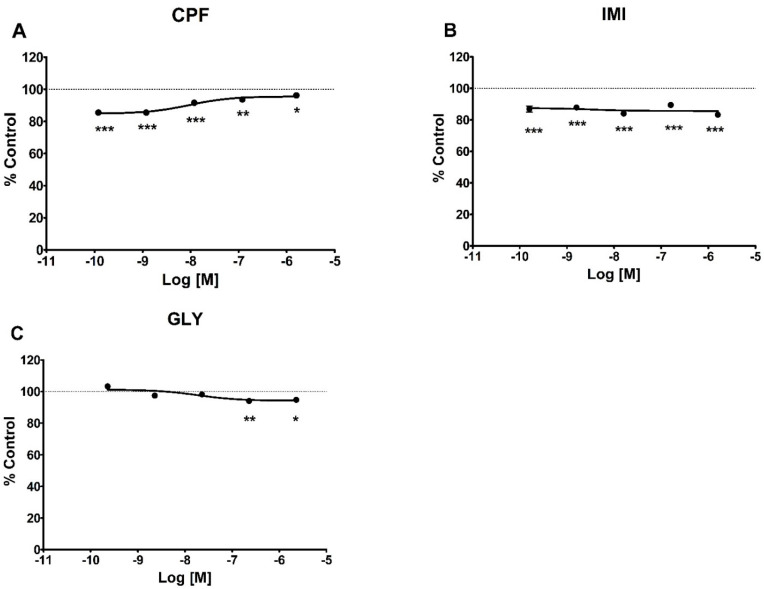
ATP levels in MCF-7 cells treated for 72 h with CPF, (**A**) IMI (**B**) or GLY (**C**). Values are means ± SEM of three independent experiments with control cells set at 100%. Asterisks indicate statistically significant differences with respect to control cells: * *p* < 0.05; ** *p* < 0.01; *** *p* < 0.001.

In MCF-7 cells (Figure 4), all three pesticides induced a decrease in intracellular ATP levels but with different patterns: a comparable and significant decrease at all concentrations for IMI, a concentration-dependent decrease for GLY significant at the two highest concentrations (230 nM and 2.3 µM), and an inverse dose-related effect for CPF significant at all concentrations tested. The pesticides did not affect intracellular ATP levels in MCF-12A cells (Figure S3).

### 3.4. Reactive Oxygen Species

**Figure 5:**
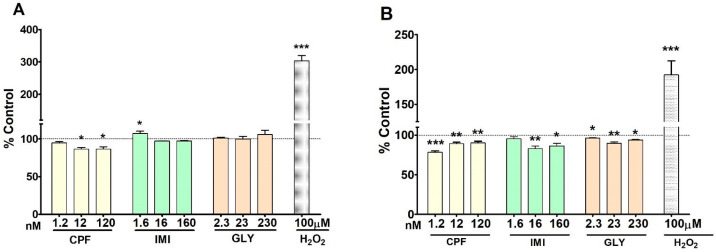
Intracellular ROS production in MCF-7 cells (**A**) and MCF-12A cells (**B**) treated with CPF, IMI, GLY or H_2_O_2_ as a positive control. Values are means ± SEM of three independent experiments with control cells set at 100%. Asterisks indicate statistically significant differences with respect to control cells: * *p* < 0.05; ** *p* < 0.01; *** *p* < 0.001.

CPF, IMI and GLY differently affected intracellular ROS production after 24 h of exposure in MCF-7 and MCF-12A cell lines, as shown in Figure 5A,B, respectively. Treatment of MCF-7 cells with CPF at 12 and 120 nM induced a significant decrease of ROS production, whereas IMI at 1.6 nM induced a significant increase. GLY induced no effects at any tested concentration. In MCF-12A cells, a statistically significant reduction in intracellular ROS species was observed following treatment with CPF and GLY at all concentrations, and at the two highest concentrations of IMI. As expected, the positive control, H_2_O_2_ 100 mM, caused a strong significant increase of intracellular ROS levels in both cell lines.

### 3.5. Estradiol Secretion

**Figure 6:**
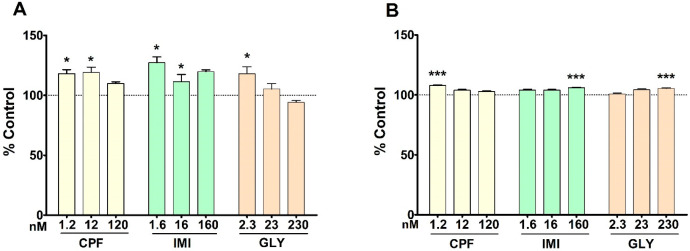
E2 secretion in MCF-7 cells (**A**) and MCF-12A cells (**B**) following treatment with CPF, IMI and GLY or medium alone as a control, for 72 h. Values are means ± SEM of three independent experiments with control cells set at 100%. Asterisks indicate statistically significant differences with respect to control cells: * *p* < 0.05; *** *p* < 0.001.

E2 secretion was generally increased in both cell lines by the three pesticides. In MCF-7 cells induction was observed upon treatment with CPF at 1.2 nM and 12 nM, IMI at 1.6 and 16 nM and GLY at 2.3 nM (Figure 6A).

In MCF-12A cells, E2 secretion was increased by CPF at the lowest concentration, but to a lower extent compared to MCF-7 cells, and by IMI and GLY at the highest concentrations (Figure 6B).

### 3.6. Nuclear Receptors’ Gene Expression

**Figure 7:**
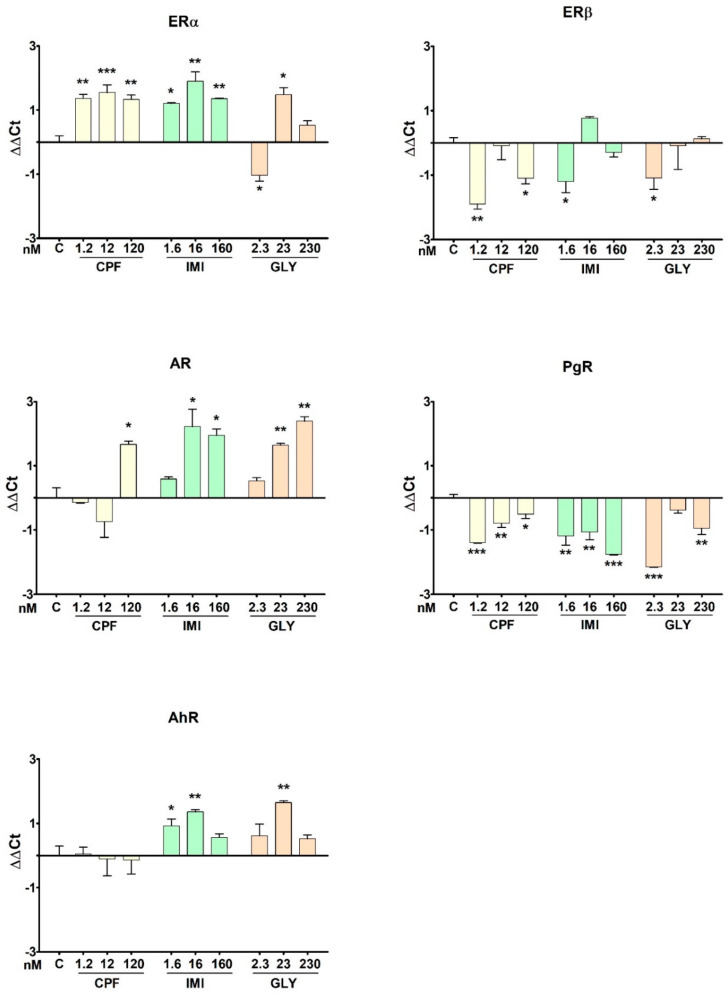
Gene expression levels of a panel of nuclear receptors assessed in MCF-7 cells following treatment with CPF, IMI and GLY for 72 h. Data are mean ΔΔCt values ± SEM of three independent experiments, with control cells as calibrators and the geometric mean of GAPDH and ACTB as a reference. Asterisks indicate statistically significant differences with respect to control cells: * *p* < 0.05; ** *p* < 0.01; *** *p* < 0.001.

The gene expression levels of the selected NRs (i.e., ERα, ERβ, AR, AhR and PgR) in MCF-7 cells are shown in Figure 7. ERα gene expression was significantly up-regulated by CPF and IMI at all tested concentrations and by GLY at the intermediate concentration (23 nM). Conversely, GLY at 2.3 nM significantly down-regulated ERα expression. ERβ gene expression was repressed by CPF at the lowest and highest doses (1.2 and 120 nM) and by IMI and GLY at the lowest doses (1.6 nM and 2.3 nM, respectively).

The expression of AR was dose-dependently increased by GLY, being significant at the two highest doses (23 and 230 nM), by IMI at the two highest doses (16 and 160 nM) and by CPF at 120 nM.

PgR gene expression was significantly down-regulated by CPF and IMI at all tested concentrations, and by GLY at the intermediate dose only (23 nM). AhR gene expression was induced following treatment with IMI at the low and intermediate concentrations (1.6 nM and 16 nM) and with GLY at the intermediate concentration (23 nM); no effect on AhR was exerted by CPF.

**Figure 8:**
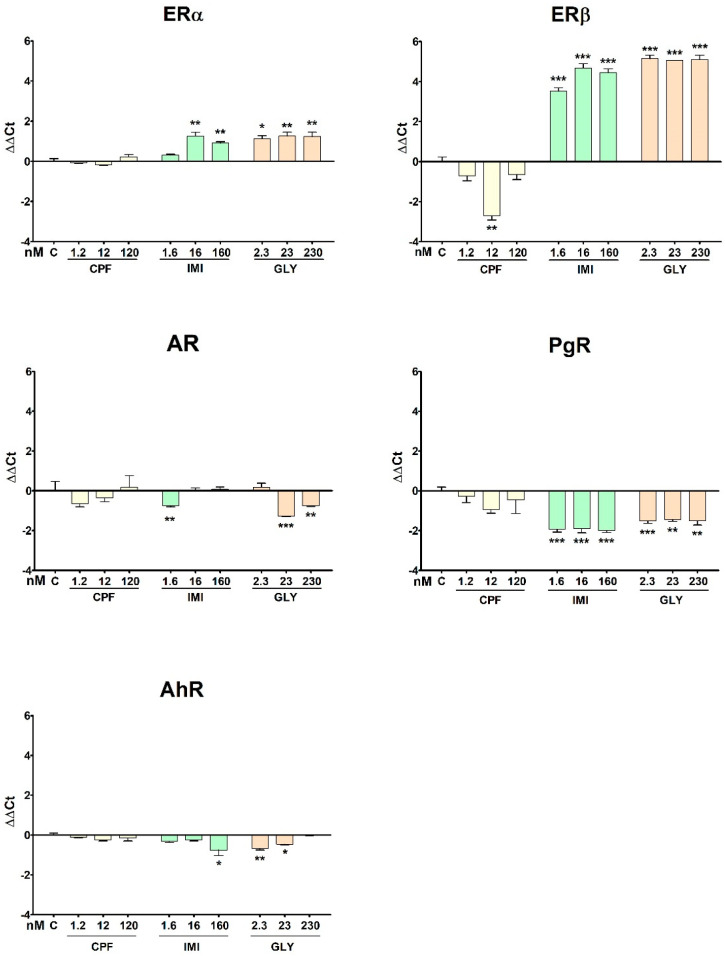
Gene expression levels of a panel of nuclear receptors assessed in MCF-12A cells following treatment with CPF, IMI and GLY for 72 h. Data are mean ΔΔCt values ± SEM of three independent experiments, with control samples as calibrators and the geometric mean of GAPDH and ACTB as reference. Asterisks indicate statistically significant differences with respect to control cells: * *p* < 0.05; ** *p* < 0.01; *** *p* < 0.001.

The effects of the three pesticides on the NRs expression in MCF-12A are shown in Figure 8. ERα was significantly up-regulated by IMI at 16 and 160 nM and by GLY at all tested concentrations, whereas no effect was observed for CPF. ERβ was down-regulated by CPF at the intermediate concentration (16 nM), whereas it was significantly up-regulated by IMI and GLY at all tested concentrations. AR gene expression was repressed by IMI at the lowest dose (1.6 nM) and by GLY at the two highest doses (23 and 230 nM). No significant effect was observed upon CPF treatment.

The expression of PgR was down-regulated by IMI and GLY at all concentrations tested. AhR gene expression was decreased by GLY at the lower and intermediate concentrations (2.3 and 23 nM) and by IMI at the highest dose (160 nM). No significant effect was exerted by CPF on PgR and AhR expression.

## 4. Discussion

The present study evaluated the toxicological effects of CPF, IMI and GLY on human breasts in in vitro models by comparing the responses related to several toxicological endpoints possibly involved in mammary gland development, in the widely used MCF-7 tumorigenic cell line, and in the MCF-12A non-tumorigenic cell line, as a representative of normal physiological conditions. For a more realistic assessment, pesticide concentrations used in this study were derived from those really occurring in children [31,32,33,34,35], coincident with the nanomolar concentrations in our experiments (i.e., CPF 12 nM; IMI 16 nM; GLY 23 nM).

Among the available human non-tumorigenic breast cell lines, we selected MCF-12A because of its capability to express both ERs, as previously reported [37]. Our study confirmed such expression, moreover demonstrating that MCF-12A cells also express AR, PgR and AhR.

The three pesticides differently affected cell proliferation and cell vitality of MCF-7 and MCF-12A cell lines, with MCF-7 mainly displaying a drop in cell vitality and MCF-12A in cell proliferation. In MCF-7 cells, effects on cell proliferation were only marginal, with a slight increase at the CPF higher dose (1.2 µM) and a decrease at the GLY lower concentration (230 pM). These effects were further supported by a lack of induction in apoptotic and necrotic signals. At least for CPF, a previous induction of cell proliferation was observed in MCF-7 at 50 nM but only for long (10 days) and not for short exposure times (24 to 96 h) [21] On the contrary, GLY induced proliferation in MCF-7 cells at higher concentrations >500 µM [6] than those tested in this study.

In MCF-12A, CPF and GLY both induced a dose-dependent decrease in cell proliferation, but GLY repression was more severe, with a drop of about 28% at the highest dose (2.3 µM). Accordingly, GLY was the only pesticide inducing early apoptosis, in these cells at 7 and 8 h, although only at 2.3 nM concentration. Noteworthily, the proliferation decrease induced by CPF occurred also in the nano- and picomolar ranges, suggesting a different mechanism for CPF and a high responsiveness of MCF-12A cells to this endpoint.

IMI also decreased cell proliferation in MCF-12A cells but only at the highest dose (1.6 µM). In an E-screen test using MCF-7 cells, IMI inhibited cell proliferation at a concentration corresponding to about 195 µM [38], which is 100-fold higher than our highest dose; this evidence further supports MCF-12A as more responsive for cell proliferation assessment.

Interestingly, CPF and IMI induced a slight decrease in apoptotic signal, suggesting that these pesticides may have triggered a different cell death process leading to the observed cell proliferation inhibition.

Cell vitality was dose-dependently decreased by all three pesticides in MCF-7 cells, with the more striking effect exerted by CPF reducing vitality by about 28% at the highest dose. CPF repressed cell vitality also in MCF-12A cells, whereas the two other pesticides did not significantly affect it. Thus, MCF-7 cells appear more sensitive to metabolic derangement compared to MCF-12A. Indeed, only in MCF-7 we observed a fall in ATP intracellular levels induced by all three pesticides, whereas no effect was observed in MCF-12A. Such observations support previous evidence comparing MCF-7 and MCF-12A response to oxidative stress and mitochondrial impairment affecting only MCF-7 tumorigenic cells due to their aberrant mitochondria functions, which cause a lack of competence in controlling stress conditions [39].

The ATP depletion in MCF-7 cells was not generally supported by a concomitant increase in ROS levels. Rather, CPF induced a decrease of ROS production at 12 and 120 nM. A similar effect was observed also in MCF-12A cells where intracellular ROS were decreased by all three pesticides. Such effect may be suggestive of an autophagy induction, as previously observed [39].

Several pieces of evidence reported the ability of the three pesticides to deplete ATP synthesis, concomitant or not with an ROS increase. In dopaminergic neural cells and induced pluripotent stem cells, micromolar CPF impaired mitochondrial membrane potential with a consequent reduction in ATP [40,41]; an ROS increase was also observed [40]. In A549, CPF induced intracellular ROS at 10 and 100 nM, decreasing cell vitality only at micromolar concentrations [42]. In the same range of concentrations, CPF induced morphological alterations of mitochondria in HeLa cells as well as ATP depletion in SH-SY5Y cells [43]. Decreased ATP production was also observed upon IMI treatment of isolated rat liver mitochondria in the micromolar range, without affecting mitochondrial membrane potential [44]. No increase in ROS production was observed in lymphoblastoid cells treated with IMI up to 391 nM [45]. GLY at 0.036 g/L increased intracellular Ca^2+^ concentrations in rat Sertoli cells [46] due to its ability to enhance mitochondrial membrane permeability, which finally leads to decreased ATP synthesis [47]. Inhibition of ATP synthesis following GLY exposure was observed also in human peripheral blood mononuclear cells, although in the millimolar range [48], and in swine granulosa cells in the micromolar range [49]. In female adult mice administered 250 or 500 mg/kg bw GLY, ATP decrease was observed in the ovary, with a concurrent increase in ROS levels and decrease of the mitochondrial membrane potential [50].

Thus, although the available evidence indicates that the three pesticides differently affect the mitochondrial membrane potential/permeability, all impaired ATP synthesis in MCF-7 but not in MCF-12A; at high exposure concentrations this effect may translate into an increase of ROS production, not observed in our experiment performed at human relevant concentrations. How this could affect mammary gland development has to be further investigated.

As regards endocrine endpoints, we observed that the three pesticides under study differently affected E2 secretion and gene expression of NRs involved in mammary gland development. To our knowledge, this is the first time that CPF, IMI and GLY effects were simultaneously assessed on this panel of NRs in both MCF-7 and MCF-12A cell lines.

All three pesticides increased E2 secretion in both cell lines, although to a different extent. This effect was generally supported by an up-regulation of ERα gene expression in both cell lines but with some exceptions; indeed, in MCF-7 cells, ERα was induced by CPF and IMI, whereas GLY downregulated its expression at the lowest dose and induced it at the intermediate dose. In MCF-12A cells, ERα was up-regulated by IMI and GLY whereas CPF did not affect its expression. Noteworthily, all three pesticides downregulated ERβ in MCF-7 cells whereas a strong expression induction was observed in MCF-12A cells upon IMI and GLY treatment at all doses; on the contrary, CPF repressed ERβ also in MCF-12A cells. Thus, a different balance of ERα and ERβ receptors may occur following treatment with these pesticides, even at these human relevant concentrations, with possible consequences on the proper mammary gland development.

PgR gene expression was repressed by all pesticides in MCF-7 and by IMI and GLY in MCF-12A. In MCF-7, all three pesticides up-regulated AR whereas only IMI and GLY upregulated AhR. These same pesticides downregulated AR and AhR also in MCF-12A although to a lesser extent. No effect was induced by CPF on these two receptors in MCF-12A cells.

Overall, these results support dissimilar mechanism underlying the action of each pesticide also in relation to the tumorigenic or non-tumorigenic origin of the cell line considered, which may differ in co-activators’/co-repressors’ abundances. Of note, CPF almost did not affect NRs gene expression in MCF-12A, except for the downregulation of ERβ, at least in the range of concentrations tested.

Previous evidence on CPF reported decreased ERα gene expression in MCF-7 cells after short (24 h) and long (14 days) exposure times at 10 and 100 µM, with corresponding protein increasing at lower exposure concentrations (0.1–10 µM). A parallel dose-related increase in AhR gene expression was observed after 14 days of exposure but not at 24 h [22]. Thus, AhR activation appeared delayed compared to ERα, which may explain the lack of effect observed in our study. In addition, tested concentrations were about 100- and 1000-fold higher than those used in our experiments, performed at shorter time of exposure.

In transactivation assays using different cell lines, CPF induced both agonistic and antagonistic activity toward ER [51,52,53] and AhR [20,53,54], no androgenic or anti-androgenic effects [18,51,53] and antagonistic activity versus PgR [53]; CPF agonism was effective only versus ERα and not ERβ [52], thus mostly confirming our observations in MCF-7 cells but not in MCF-12A cells, where CPF affected only ERβ expression.

Induction of ERβ gene expression was observed in MCF-7-BUS cells treated with micromolar CPF [16]. The observed down-regulation of ERβ upon CPF treatment of MCF-7 and MCF-12A cells suggests that a different mechanism may occur at lower concentrations.

CPF may alter mammary gland morphology, as observed in adult female rats administered with 0.01 mg and 1 mg/kg bw/day in which increased numbers of ducts and alveolar structures occurred; the effect was associated with increased PgR expression and decreased E2 serum levels with no effect on ERα expression [23]. Increased branching of ducts and higher diameter and number of buds were observed in adult female rats administered with 0.1 and 2.5 mg/kg bw/day CPF [24]. Although the endocrine effects described do not overlap with our results, dose differences and species-specific effects may explain the discrepancy. In any case, our results support CPF as a potential risk factor for mammary gland development due to the estrogenic activity observed in both MCF-7 and MCF-12A cells.

Limited evidence is available for IMI endocrine disrupting activity. In particular, estrogenic activity was observed in transfected human breast cancer cells (MELN) in the micromolar range [26], whereas no androgenic activity was detected [55]. A significant E2 reduction was observed in female rats administered 50–300 mg/kg bw/day IMI [56]. No effect on AhR expression was observed in the livers of mice treated with 0.6 mg/kg bw/day [57]. Our results do not overlap completely with available literature, since we observed both AR and AhR induction by IMI in MCF-7 cells. However, previous evidence supports the observed increase of E2 secretion only at the lowest concentration tested, as well as the induction of ERα gene expression. No evidence for IMI effects on ERβ and PgR receptors is available to compare with; however, in our experimental model, IMI displayed opposing effects on ERβ expression in the two cells lines, also repressing PgR in both cells. Thus, since IMI may affect mammary gland development deranging the expression of these endocrine endpoints, further studies investigating IMI effects on this tissue are warranted.

We observed that GLY and IMI exerted somewhat similar effects, especially in MCF-12A cells. Previous evidence on GLY reported activation of estrogen responsive elements in hormone-dependent breast cancer cells (T47D) in the nano- and micromolar ranges [6,28]; both ERs gene expression was induced by 1 and 100 nM GLY at 6 h but not at 24 h [28], thus suggesting an early triggering effect. Conversely, no ER transactivation was observed in other transfected cells [26,27]. However, GLY displayed anti-androgenic activity [27] and strongly inhibited aromatase activity [26] in the micromolar range. Available evidence of in vivo studies is contrasting as regards GLY effects on NRs. Postnatal administration of a GLY-based formulation (2 mg/kg bw/day GLY) to female rats affected mammary gland development, inducing an increase of hyperplastic ducts displaying enhanced ERα and PgR protein expression [29]. On the contrary, PgR decreased expression was observed in the uterus of developmentally exposed female rats with a concomitant increase of ERα expression and E2 serum levels [58]. Our results further support a concern for GLY adverse effects on mammary gland, also at low human relevant exposure concentrations.

During puberty, the mammary gland development is promoted by estrogen and progesterone hormones, which, by activating ERα and PgR, induce ductal elongation and alveolar formation, whereas ERβ promotes apoptosis and differentiation of the epithelial tissue [59,60]. The action of AR and AhR is also important, as AR counteracts the extent of proliferation, ductal extension and bud morphology [13], and AhR, due to cross-talk with ERs and coactivator of ERα, regulates proliferation [15]. In addition, E2 secretion, which can be performed locally by aromatase, may further activate estrogenic pathways including vascular endothelial grow factor transcription [61], and thus mammary gland angiogenesis.

The process may occur even if PgR is downregulated. Indeed, studies in mice lacking PgR demonstrated that females had normal glands at puberty but no alveolar formation at lactation [12], thus indicating a more relevant role of PgR during a later phase of mammary gland development [62,63].

The three pesticides under study, at concentrations really occurring in children, differently affected cell proliferation, metabolic activity and endocrine biomarkers involved in mammary gland development in both MCF-7 and MCF-12A cells, suggesting a potential ability to affect molecular processes in this organ even at low doses with deranging effects on its functionality.

The present results prompt further investigations to better clarify the different mechanisms underlying pesticides effects.

## 5. Conclusions

This is the first study providing evidence on toxicological effects of CPF, IMI and GLY on both tumorigenic (MCF-7) and non-tumorigenic (MCF-12A) human mammary cell lines. The three pesticides exerted non-overlapping effects on the panel of endpoints evaluated at concentrations really occurring in children, all displaying endocrine disrupting activity. Although no definitive conclusion could be drawn regarding outcomes on mammary gland development, the results raise the concern regarding possible adverse effects induced by exposure to these compounds, especially in vulnerable population groups such as children. Both cell lines proved to be suitable and sensitive models differently highlighting the effects of pesticides; in particular, the MCF-12A cell line can be considered a valid model to evaluate the potential effects of endocrine disrupting chemicals as representative of physiological cell conditions.

## Figures and Tables

**Table 1:** Sequences of the specific primers used in real-time PCR analysis with accession numbers of the corresponding genes.

Gene	RefSeq Accession		Sequence 5′ to 3′
GAPDH	NM_002046.4	fw	ACTCCTCCACCTTTGACGCT
		rev	CTTCAAGGGGTCTACATGGC
ACTB	NM_001101.5	fw	CAGCAAGCAGGAGTATGACG
		rev	GTGAACTTTGGGGGATGCTC
ERα	NM_000125.3	fw	ACTGCGGGCTCTACTTCATC
		rev	GGCTGTTCCCAACAGAAGAC
ERβ	NM_001040275.1	fw	CTCTTTTGCCTGAAGCAACG
		rev	CTGGGCAGTTAAGGAGACCA
AR	NM_000044.3	fw	CCCATCTATTTCCACACCCA
		rev	GCAAAGTCTGAAGGTGCCAT
AhR	NM_001621.4	fw	TTCCACCTCAGTTGGCTTTG
		rev	GGACTCGGCACAATAAAGCA
PgR	NM_000926.4	fw	AGGTCTACCCGCCCTATCTC
		rev	AGTAGTTGTGCTGCCCTTCC

## Supplementary Materials

The following supporting information can be downloaded at: https://www.mdpi.com/article/10.3390/ijerph19084453/s1, Figure S1: Apoptotic and necrotic signals evaluated by Annexin V assay in MCF-7 cells treated with CPF (A,B), IMI (C,D) and GLY (E,F) or medium alone as control for 24 h. Values are fold change of three independent experiments; Figure S2: Necrotic signals evaluated by Annexin V assay in MCF-7 cells treated with CPF (A), IMI (B) and GLY (C) or medium alone as control for 24 h. Values are fold change of three independent experiments; Figure S3: ATP levels in MCF-12A cells treated for 72 h with CPF (A) IMI (B) or GLY (C). Values are means ± SEM of three independent experiments with control cells set at 100%.

## References

[1] Aktar M.W., Chowdhury A. (2009). Impact of Pesticides use in Agriculture: Their Benefits and Hazards. Interdiscip. Toxicol..

[2] Kim K., Kabir E., Jahan S.A. (2017). Exposure to Pesticides and the Associated Human Health Effects. Sci. Total Environ..

[3] Encarnação T., Pais A.A., Campos M.G., Burrows H.D. (2019). Endocrine Disrupting Chemicals: Impact on Human Health, Wildlife and the Environment. Sci. Prog..

[4] European Parliament (2009). Directive 2009/128/EC of the European Parliament and of the Council of 21 October 2009 Establishing a Framework for Community Action to Achieve the Sustainable Use of Pesticides.

[5] Andersson N., Arena M., Auteri D., Barmaz S., Grignard E., Kienzler A., Lepper P., Lostia A.M., Munn S., European Chemical Agency (ECHA) and European Food Safety Authority (EFSA) with the technical support of the Joint Research Centre (JRC) (2018). Guidance for the Identification of Endocrine Disruptors in the Context of Regulations (EU) no 528/2012 and (EC) no 1107/2009. EFSA J..

[6] Mesnage R., Phedonos A., Biserni M., Arno M., Balu S., Corton J.C., Ugarte R., Antoniou M.N. (2017). Evaluation of Estrogen Receptor Alpha Activation by Glyphosate-Based Herbicide Constituents. Food Chem. Toxicol..

[7] Rossetti M.F., Stoker C., Ramos J.G. (2020). Agrochemicals and Neurogenesis. Mol. Cell. Endocrinol..

[8] Mostafalou S., Abdollahi M. (2017). Pesticides: An Update of Human Exposure and Toxicity. Arch. Toxicol..

[9] Bianchi S., Nottola S.A., Torge D., Palmerini M.G., Necozione S., Macchiarelli G. (2020). Association between Female Reproductive Health and Mancozeb: Systematic Review of Experimental Models. Int. J. Environ. Res. Public Health.

[10] Greenspan L.C., Lee M.M. (2018). Endocrine Disrupters and Pubertal Timing. Curr. Opin. Endocrinol. Diabetes Obes..

[11] Farello G., Altieri C., Cutini M., Pozzobon G., Verrotti A. (2019). Review of the Literature on Current Changes in the Timing of Pubertal Development and the Incomplete Forms of Early Puberty. Front. Pediatr..

[12] Macias H., Hinck L. (2012). Mammary Gland Development. Wiley Interdiscip. Rev. Dev. Biol..

[13] Gao Y.R., Walters K.A., Desai R., Zhou H., Handelsman D.J., Simanainen U. (2014). Androgen Receptor Inactivation Resulted in Acceleration in Pubertal Mammary Gland Growth, Upregulation of ERα Expression, and Wnt/Β-Catenin Signaling in Female Mice. Endocrinology.

[14] Le Provost F., Riedlinger G., Hee Yim S., Benedict J., Gonzalez F.J., Flaws J., Hennighausen L. (2002). The Aryl Hydrocarbon Receptor (AhR) and its Nuclear Translocator (Arnt) are Dispensable for Normal Mammary Gland Development but are Required for Fertility. Genesis.

[15] Ohtake F., Takeyama K., Matsumoto T., Kitagawa H., Yamamoto Y., Nohara K., Tohyama C., Krust A., Mimura J., Chambon P. (2003). Modulation of Oestrogen Receptor Signalling by Association with the Activated Dioxin Receptor. Nature.

[16] Grünfeld H., Bonefeld-Jorgensen E. (2004). Effect of in Vitro Estrogenic Pesticides on Human Oestrogen Receptor A and Β mRNA Levels. Toxicol. Lett..

[17] Venerosi A., Tait S., Stecca L., Chiarotti F., De Felice A., Cometa M.F., Volpe M.T., Calamandrei G., Ricceri L. (2015). Effects of Maternal Chlorpyrifos Diet on Social Investigation and Brain Neuroendocrine Markers in the Offspring–a Mouse Study. Environ. Health.

[18] Viswanath G., Chatterjee S., Dabral S., Nanguneri S.R., Divya G., Roy P. (2010). Anti-Androgenic Endocrine Disrupting Activities of Chlorpyrifos and Piperophos. J. Steroid Biochem. Mol. Biol..

[19] De Angelis S., Tassinari R., Maranghi F., Eusepi A., Di Virgilio A., Chiarotti F., Ricceri L., Pesciolini A.V., Gilardi E., Moracci G. (2009). Developmental Exposure to Chlorpyrifos Induces Alterations in Thyroid and Thyroid Hormone Levels without Other Toxicity Signs in Cd1 Mice. Toxicol. Sci..

[20] Takeuchi S., Iida M., Yabushita H., Matsuda T., Kojima H. (2008). In Vitro Screening for Aryl Hydrocarbon Receptor Agonistic Activity in 200 Pesticides using a Highly Sensitive Reporter Cell Line, DR-EcoScreen Cells, and in Vivo Mouse Liver Cytochrome P450-1A Induction by Propanil, Diuron and Linuron. Chemosphere.

[21] Ventura C., Núñez M., Miret N., Lamas D.M., Randi A., Venturino A., Rivera E., Cocca C. (2012). Differential Mechanisms of Action are Involved in Chlorpyrifos Effects in Estrogen-Dependent Or-Independent Breast Cancer Cells Exposed to Low or High Concentrations of the Pesticide. Toxicol. Lett..

[22] Moyano P., García J., García J.M., Pelayo A., Muñoz-Calero P., Frejo M.T., Anadon M.J., Lobo M., Del Pino J. (2020). Chlorpyrifos-Induced Cell Proliferation in Human Breast Cancer Cell Lines Differentially Mediated by Estrogen and Aryl Hydrocarbon Receptors and KIAA1363 Enzyme After 24 H and 14 Days Exposure. Chemosphere.

[23] Ventura C., Nieto M.R.R., Bourguignon N., Lux-Lantos V., Rodriguez H., Cao G., Randi A., Cocca C., Núñez M. (2016). Pesticide Chlorpyrifos Acts as an Endocrine Disruptor in Adult Rats Causing Changes in Mammary Gland and Hormonal Balance. J. Steroid Biochem. Mol. Biol..

[24] Nishi K., Hundal S.S. (2013). Chlorpyrifos Induced Toxicity in Reproductive Organs of Female Wistar Rats. Food Chem. Toxicol..

[25] Zárate L.V., Pontillo C.A., Español A., Miret N.V., Chiappini F., Cocca C., Álvarez L., de Pisarev D.K., Sales M.E., Randi A.S. (2020). Angiogenesis Signaling in Breast Cancer Models is Induced by Hexachlorobenzene and Chlorpyrifos, Pesticide Ligands of the Aryl Hydrocarbon Receptor. Toxicol. Appl. Pharmacol..

[26] Zhang C., Schilirò T., Gea M., Bianchi S., Spinello A., Magistrato A., Gilardi G., Di Nardo G. (2020). Molecular Basis for Endocrine Disruption by Pesticides Targeting Aromatase and Estrogen Receptor. Int. J. Environ. Res. Public Health.

[27] Gasnier C., Dumont C., Benachour N., Clair E., Chagnon M., Séralini G. (2009). Glyphosate-Based Herbicides are Toxic and Endocrine Disruptors in Human Cell Lines. Toxicology.

[28] Thongprakaisang S., Thiantanawat A., Rangkadilok N., Suriyo T., Satayavivad J. (2013). Glyphosate Induces Human Breast Cancer Cells Growth via Estrogen Receptors. Food Chem. Toxicol..

[29] Zanardi M.V., Schimpf M.G., Gastiazoro M.P., Milesi M.M., Muñoz-de-Toro M., Varayoud J., Durando M. (2020). Glyphosate-Based Herbicide Induces Hyperplastic Ducts in the Mammary Gland of Aging Wistar Rats. Mol. Cell. Endocrinol..

[30] Coppola L., Tait S., Ciferri L., Frustagli G., Merola C., Perugini M., Fabbrizi E., La Rocca C. (2020). Integrated Approach to Evaluate the Association between Exposure to Pesticides and Idiopathic Premature Thelarche in Girls: The PEACH Project. Int. J. Mol. Sci..

[31] Knudsen L.E., Hansen P.W., Mizrak S., Hansen H.K., Mørck T.A., Nielsen F., Siersma V., Mathiesen L. (2017). Biomonitoring of Danish School Children and Mothers Including Biomarkers of PBDE and Glyphosate. Rev. Environ. Health.

[32] Curwin B.D., Hein M.J., Sanderson W.T., Striley C., Heederik D., Kromhout H., Reynolds S.J., Alavanja M.C. (2007). Pesticide Dose Estimates for Children of Iowa Farmers and Non-Farmers. Environ. Res..

[33] Sierra-Diaz E., Celis-de la Rosa A.J., Lozano-Kasten F., Trasande L., Peregrina-Lucano A.A., Sandoval-Pinto E., Gonzalez-Chavez H. (2019). Urinary Pesticide Levels in Children and Adolescents Residing in Two Agricultural Communities in Mexico. Int. J. Environ. Res. Public Health.

[34] Morgan M.K., Sheldon L.S., Croghan C.W., Jones P.A., Robertson G.L., Chuang J.C., Wilson N.K., Lyu C.W. (2005). Exposures of Preschool Children to Chlorpyrifos and its Degradation Product 3, 5, 6-Trichloro-2-Pyridinol in their Everyday Environments. J. Expo. Sci. Environ. Epidemiol..

[35] Ospina M., Wong L., Baker S.E., Serafim A.B., Morales-Agudelo P., Calafat A.M. (2019). Exposure to Neonicotinoid Insecticides in the US General Population: Data from the 2015–2016 National Health and Nutrition Examination Survey. Environ. Res..

[36] Larsson P., Engqvist H., Biermann J., Rönnerman E.W., Forssell-Aronsson E., Kovács A., Karlsson P., Helou K., Parris T.Z. (2020). Optimization of Cell Viability Assays to Improve Replicability and Reproducibility of Cancer Drug Sensitivity Screens. Sci. Rep..

[37] Marchese S., Silva E. (2012). Disruption of 3D MCF-12A Breast Cell Cultures by Estrogens–An in Vitro Model for ER-Mediated Changes Indicative of Hormonal Carcinogenesis. PLoS ONE.

[38] Mesnage R., Biserni M., Genkova D., Wesolowski L., Antoniou M.N. (2018). Evaluation of Neonicotinoid Insecticides for Oestrogenic, Thyroidogenic and Adipogenic Activity Reveals Imidacloprid Causes Lipid Accumulation. J. Appl. Toxicol..

[39] Stander X.X., Stander B.A., Joubert A.M. (2015). Synergistic Anticancer Potential of Dichloroacetate and Estradiol Analogue Exerting their Effect Via ROS-JNK-Bcl-2-Mediated Signalling Pathways. Cell. Physiol. Biochem..

[40] Singh N., Lawana V., Luo J., Phong P., Abdalla A., Palanisamy B., Rokad D., Sarkar S., Jin H., Anantharam V. (2018). Organophosphate Pesticide Chlorpyrifos Impairs STAT1 Signaling to Induce Dopaminergic Neurotoxicity: Implications for Mitochondria Mediated Oxidative Stress Signaling Events. Neurobiol. Dis..

[41] Yamada S., Kubo Y., Yamazaki D., Sekino Y., Kanda Y. (2017). Chlorpyrifos Inhibits Neural Induction via Mfn1-Mediated Mitochondrial Dysfunction in Human Induced Pluripotent Stem Cells. Sci. Rep..

[42] Thakur S., Dhiman M., Mantha A.K. (2018). APE1 Modulates Cellular Responses to Organophosphate Pesticide-Induced Oxidative Damage in Non-Small Cell Lung Carcinoma A549 Cells. Mol. Cell. Biochem..

[43] Chen T., Tan J., Wan Z., Zou Y., Kessete Afewerky H., Zhang Z., Zhang T. (2017). Effects of Commonly used Pesticides in China on the Mitochondria and Ubiquitin-Proteasome System in Parkinson’s Disease. Int. J. Mol. Sci..

[44] Bizerra P.F., Guimarães A.R., Maioli M.A., Mingatto F.E. (2018). Imidacloprid Affects Rat Liver Mitochondrial Bioenergetics by Inhibiting FoF1-ATP Synthase Activity. J. Toxicol. Environ. Health A.

[45] Guo J., Shi R., Cao Y., Luan Y., Zhou Y., Gao Y., Tian Y. (2020). Genotoxic Effects of Imidacloprid in Human Lymphoblastoid TK6 Cells. Drug Chem. Toxicol..

[46] de Liz Oliveira Cavalli V.L., Cattani D., Heinz Rieg C.E., Pierozan P., Zanatta L., Benedetti Parisotto E., Wilhelm Filho D., Mena Barreto Silva F.R., Pessoa-Pureur R., Zamoner A. (2013). Roundup Disrupts Male Reproductive Functions by Triggering Calcium-Mediated Cell Death in Rat Testis and Sertoli Cells. Free Radic. Biol. Med..

[47] Olorunsogo O.O. (1990). Modification of the Transport of Protons and Ca2 Ions across Mitochondrial Coupling Membrane by N-(Phosphonomethyl) Glycine. Toxicology.

[48] Kwiatkowska M., Jarosiewicz P., Michałowicz J., Koter-Michalak M., Huras B., Bukowska B. (2016). The Impact of Glyphosate, its Metabolites and Impurities on Viability, ATP Level and Morphological Changes in Human Peripheral Blood Mononuclear Cells. PLoS ONE.

[49] Gigante P., Berni M., Bussolati S., Grasselli F., Grolli S., Ramoni R., Basini G. (2018). Glyphosate Affects Swine Ovarian and Adipose Stromal Cell Functions. Anim. Reprod. Sci..

[50] Zhang J., Zhao C., Shi F., Zhang S., Wang S., Feng X. (2021). Melatonin Alleviates the Deterioration of Oocytes and Hormonal Disorders from Mice Subjected to Glyphosate. Mol. Cell. Endocrinol..

[51] Andersen H.R., Vinggaard A.M., Rasmussen T.H., Gjermandsen I.M., Bonefeld-Jørgensen E.C. (2002). Effects of Currently used Pesticides in Assays for Estrogenicity, Androgenicity, and Aromatase Activity in Vitro. Toxicol. Appl. Pharmacol..

[52] Kojima H., Katsura E., Takeuchi S., Niiyama K., Kobayashi K. (2004). Screening for Estrogen and Androgen Receptor Activities in 200 Pesticides by in Vitro Reporter Gene Assays using Chinese Hamster Ovary Cells. Environ. Health Perspect..

[53] Doan T., Connolly L., Igout A., Nott K., Muller M., Scippo M. (2020). In Vitro Profiling of the Potential Endocrine Disrupting Activities Affecting Steroid and Aryl Hydrocarbon Receptors of Compounds and Mixtures Prevalent in Human Drinking Water Resources. Chemosphere.

[54] Long M., Laier P., Vinggaard A.M., Andersen H.R., Lynggaard J., Bonefeld-Jørgensen E.C. (2003). Effects of Currently used Pesticides in the AhR-CALUX Assay: Comparison between the Human TV101L and the Rat H4IIE Cell Line. Toxicology.

[55] Kugathas S., Audouze K., Ermler S., Orton F., Rosivatz E., Scholze M., Kortenkamp A. (2016). Effects of Common Pesticides on Prostaglandin D2 (PGD2) Inhibition in SC5 Mouse Sertoli Cells, Evidence of Binding at the COX-2 Active Site, and Implications for Endocrine Disruption. Environ. Health Perspect..

[56] Mzid M., Ghlissi Z., Salem M.B., Khedir S.B., Chaabouni K., Ayedi F., Sahnoun Z., Hakim A., Rebai T. (2018). Chemoprotective Role of Ethanol Extract of *Urtica Urens* L. Against the Toxicity of Imidacloprid on Endocrine Disruption and Ovarian Morphometric in Female Rats, GC/MS Analysis. Biomed. Pharmacother..

[57] Nimako C., Ikenaka Y., Okamatsu-Ogura Y., Bariuan J.V., Kobayashi A., Yamazaki R., Taira K., Hoshi N., Hirano T., Nakayama S.M. (2021). Chronic Low-Dose Exposure to Imidacloprid Potentiates High Fat Diet-Mediated Liver Steatosis in C57BL/6J Male Mice. J. Vet. Med. Sci..

[58] Lorenz V., Pacini G., Luque E.H., Varayoud J., Milesi M.M. (2020). Perinatal Exposure to Glyphosate or a Glyphosate-Based Formulation Disrupts Hormonal and Uterine Milieu during the Receptive State in Rats. Food Chem. Toxicol..

[59] Stingl J. (2011). Estrogen and Progesterone in Normal Mammary Gland Development and in Cancer. Horm. Cancer.

[60] Morani A., Warner M., Gustafsson J. (2008). Biological Functions and Clinical Implications of Oestrogen Receptors Alfa and Beta in Epithelial Tissues. J. Intern. Med..

[61] Nakamura J., Lu Q., Aberdeen G., Albrecht E., Brodie A. (1999). The Effect of Estrogen on Aromatase and Vascular Endothelial Growth Factor Messenger Ribonucleic Acid in the Normal Nonhuman Primate Mammary Gland. J. Clin. Endocrinol. Metab..

[62] Arendt L.M., Kuperwasser C. (2015). Form and Function: How Estrogen and Progesterone Regulate the Mammary Epithelial Hierarchy. J. Mammary Gland Biol. Neoplasia.

[63] Brisken C., Park S., Vass T., Lydon J.P., O’Malley B.W., Weinberg R.A. (1998). A Paracrine Role for the Epithelial Progesterone Receptor in Mammary Gland Development. Proc. Natl. Acad. Sci. USA.

