# Supplementary material for: Comparison of the Toxicological Effects of Pesticides in Non-Tumorigenic MCF-12A and Tumorigenic MCF-7 Human Breast Cells
Source: Int J Environ Res Public Health. 2022 Apr 7;19(8):4453. doi: 10.3390/ijerph19084453 (PMC9030493; doi:10.3390/ijerph19084453)
Supplement: Supplementary file 1 [file ijerph-19-04453-s001.zip › ijerph-1640620-supplementary.pdf]

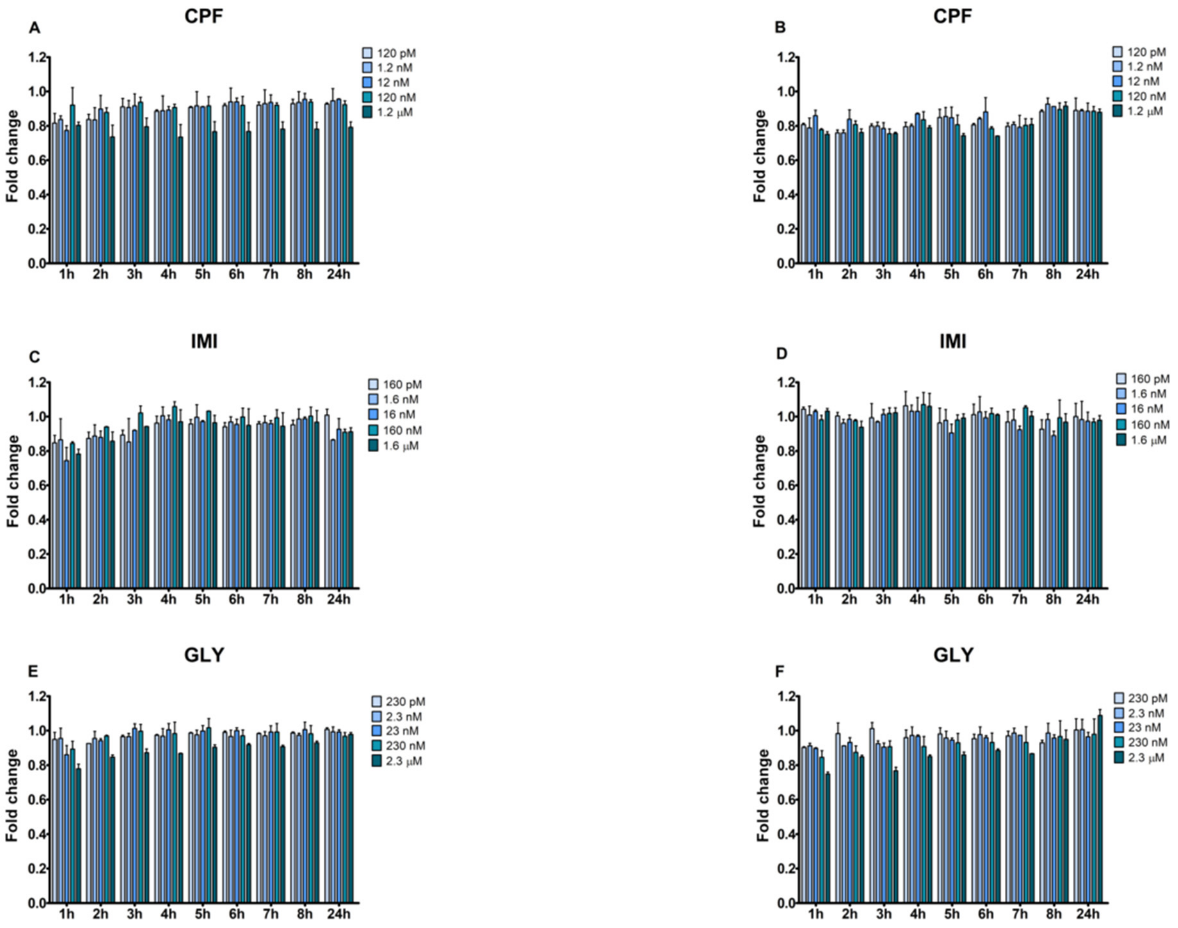

Figure S1. Apoptotic and necrotic signals evaluated by Annexin V assay in MCF-7 cells treated with CPF (**A**,**B**), IMI (**C**,**D**) and GLY (**E**,**F**) or medium alone as control for 24 h. Values are fold change of three independent experiments.

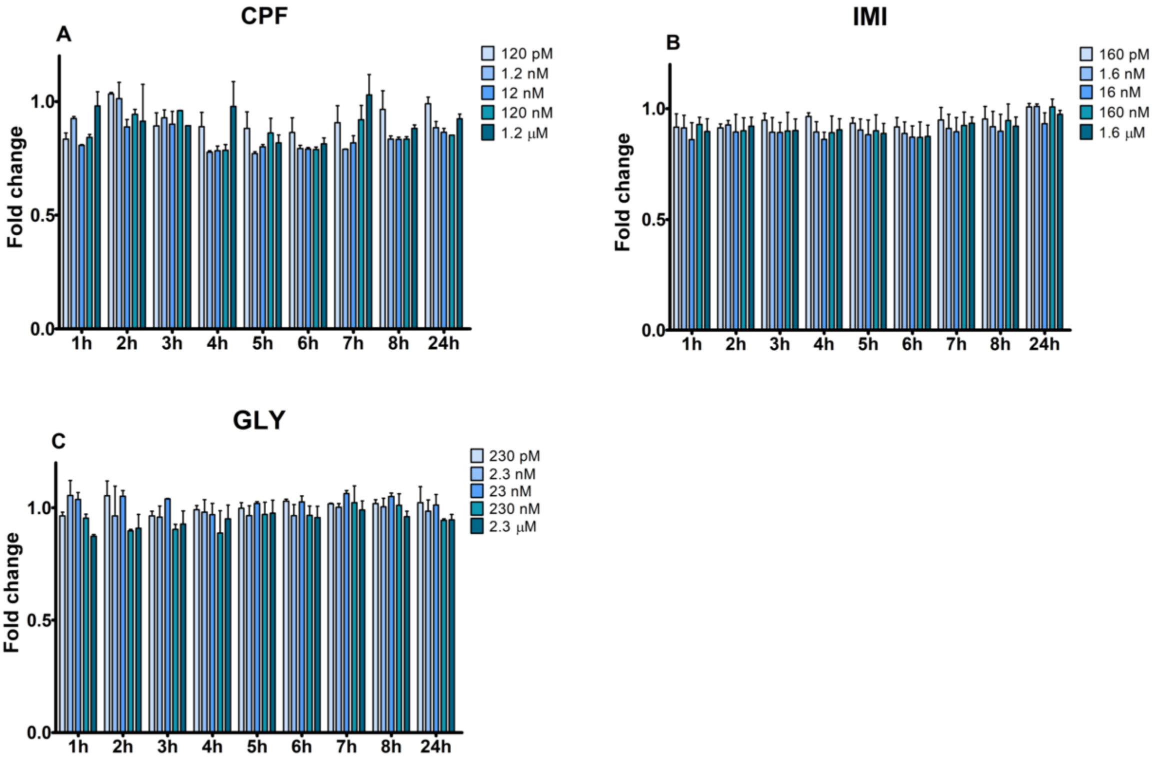

Figure S2. Necrotic signals evaluated by Annexin V assay in MCF-7 cells treated with CPF (**A**), IMI (**B**) and GLY (**C**) or medium alone as control for 24 h. Values are fold change of three independent experiments.

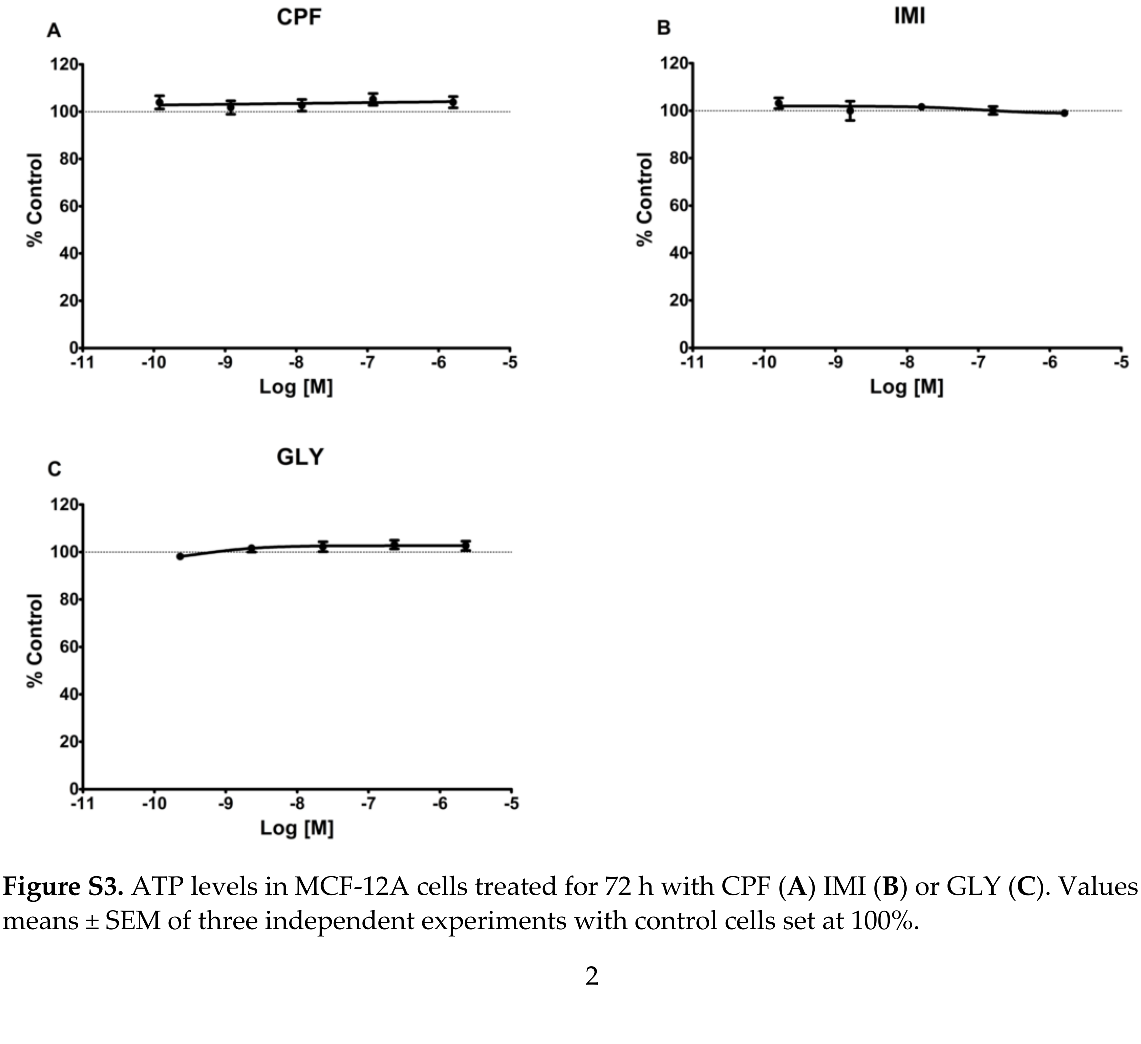

Figure S3. ATP levels in MCF-12A cells treated for 72 h with CPF (**A**) IMI (**B**) or GLY (**C**). Values are means ± SEM of three independent experiments with control cells set at 100%.
